# Comparative Analysis of GF-5 and Sentinel-2A Fusion Methods for Lithological Classification: The Tuanjie Peak, Xinjiang Case Study

**DOI:** 10.3390/s24041267

**Published:** 2024-02-16

**Authors:** Yujin Chi, Nannan Zhang, Liuyuan Jin, Shibin Liao, Hao Zhang, Li Chen

**Affiliations:** 1 Xinjiang Institute of Ecology and Geography, Chinese Academy of Sciences, Urumqi 830011, China; chiyujin20@mails.ucas.ac.cn (Y.C.); liaoshibin@ms.xjb.ac.cn (S.L.); zhanghao19@mails.ucas.ac.cn (H.Z.); chenli183@mails.ucas.ac.cn (L.C.); 2 University of Chinese Academy of Sciences, Beijing 100049, China; 3 Xinjiang Key Laboratory of Mineral Resources and Digital Geology, Urumqi 830011, China; 4 Xinjiang Research Center for Mineral Resources, Chinese Academy of Sciences, Urumqi 830011, China; 5 Geological Survey Academy of Xinjiang, Urumqi 830000, China; Jinliuyuan89@sina.com

**Keywords:** remote sensing, image fusion, lithologic classification, comparative evaluation

## Abstract

This study investigates the application of hyperspectral image space–spectral fusion technology in lithologic classification, using data from China’s GF-5 and Europe’s Sentinel-2A. The research focuses on the southern region of Tuanjie Peak in the Western Kunlun Range, comparing five space–spectral fusion methods: GSA, SFIM, CNMF, HySure, and NonRegSRNet. To comprehensively evaluate the effectiveness and applicability of these fusion methods, the study conducts a comprehensive assessment from three aspects: evaluation of fusion effects, lithologic classification experiments, and field validation. In the evaluation of fusion effects, the study uses an index analysis and comparison of spectral curves before and after fusion, concluding that the GSA fusion method performs the best. For lithologic classification, the Random Forest (RF) classification method is used, training with both area and point samples. The classification results from area sample training show significantly higher overall accuracy compared to point samples, aligning well with 1:50,000 scale geological maps. In field validation, the study employs on-site verification combined with microscopic identification and comparison of images with actual spectral fusion, finding that the classification results for the five lithologies are essentially consistent with field validation results. The “GSA+RF” method combination established in this paper, based on data from GF-5 and Sentinel-2A satellites, can provide technical support for lithological classification in similar high-altitude regions.

## 1. Introduction

Hyperspectral sensors are capable of imaging multiple target areas within the electromagnetic spectrum [1]. Due to the limitations of imaging cameras, there exists a trade-off between spectral and spatial resolution, leading to hyperspectral images often having a lower spatial resolution, especially in the majority of hyperspectral data bands concentrated in the visible light range (400–1000 nm). Hyperspectral imaging, with its high spectral resolution, finds extensive applications in fields such as facial recognition [2], medical diagnostics [3], geological exploration [4], resource and environmental surveys [5,6,7], and agricultural monitoring [8,9,10,11].

In geological applications, the focus is primarily on the short-wave infrared range (1000–2500 nm) [12]. However, the number of hyperspectral datasets that satisfy this spectral range is limited, as outlined in Table 1. Each hyperspectral satellite in the table has its drawbacks. For example, although Hypersion data are available worldwide and takes only a few hours for the first-time access (considering registration and licensing), and just a few hours under “operational mode”, it is expensive. Additionally, its narrow swath width also limits the application of this data. Currently, for Chinese users, the GF-5 hyperspectral data are more conveniently accessible and widely used due to their relative advantages in various aspects. GF-5 is equipped with visible–near-infrared (VNIR) and shortwave infrared (SWIR) sensors, which together form a crucial part of its remote sensing capabilities. The VNIR sensor is responsible for capturing data from the visible to near-infrared spectrum, playing a significant role in analyzing surface vegetation, water bodies, and urban environments. The SWIR sensor, on the other hand, focuses on the shortwave infrared spectrum, crucial for identifying and analyzing mineral compositions, soil moisture, and other surface features. GF-5, with the combination of these two sensors, can provide more comprehensive and in-depth surface information, supporting various applications such as environmental monitoring, natural resource assessment, and disaster management.

**Figure 1:**
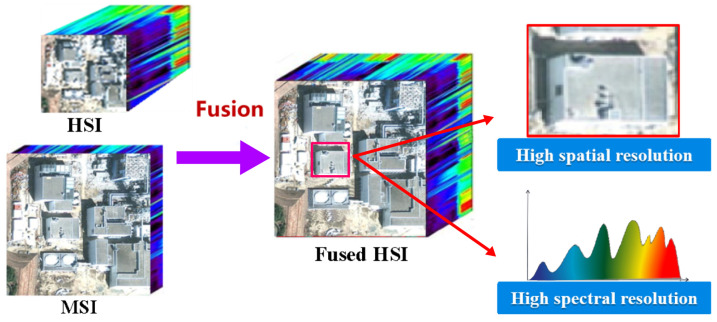
Schematic diagram of HSI-MSI fusion [20].

However, given its spatial resolution of 30 m, a key research focus in remote sensinggeology is how to fuse GF-5 data with other multispectral data of higher spatial resolution, thereby enhancing its capacity to describe spatial details and expand the scope andeffectiveness of its application. The fusion process is illustrated in Figure 1.

Data fusion represents an effective solution to the limitations faced by hyperspectral satellites in terms of temporal and spatial resolution, among other metrics [13]. Currently, the methods used for HSI-MSI fusion (hyperspectral and multispectral fusion) can be categorized into five main types [14]. These include pansharpening methods (such as Component Substitution (CS) [14] and Multi-Resolution Analysis (MRA) [15]), tensor-based methods [16], Bayesian algorithms [17], matrix decomposition algorithms [18], and deep learning approaches [19]. This classification underscores the diversity and complexity of techniques employed to enhance the quality and utility of satellite imagery data, addressing the inherent constraints of hyperspectral satellites.

Component Substitution (CS) methods, which emerged earliest, primarily focus on separating the spectral and spatial components of multispectral images. The difference between various CS methods lies in their transformation techniques. Notable among these are the Intensity–Hue–Saturation (IHS) transformation [21], Principal Components Analysis (PCA [22]), and Gram Schmidt (GS) orthogonal transformation [23]. Multi-Resolution Analysis (MRA) methods, on the other hand, involve injecting high-resolution structures obtained through multi-resolution decomposition into low-resolution structures, effectively restoring spatial details. This category includes methods such as wavelet transforms [24,25], Laplacian pyramids [26], contourlets [27], and curvelets [28]. These methodologies illustrate the nuanced approaches in enhancing the spatial and spectral resolution of satellite imagery data.

In recent years, tensor representation has become a hot research topic in HSI-MSI fusion. This can be further divided into methods based on tensor decomposition and tensor representation. Common tensor decomposition methods include CANDECOMP/PARAFAC decomposition [29,30], Tucker decomposition [31,32], Tensor Train (TT) decomposition [33], and Tensor Singular Value Decomposition (T-SVD) [34].

Within Bayesian methods, Akhtar et al. [35,36] initially proposed a Bayesian sparse representation approach. Zhang et al. [37] improved upon the Bayesian fusion model proposed in the literature [38] by assuming a joint normal model for images in the wavelet domain and adopting an additive noise imaging model and defined operators to describe the spatial degradation of LRHS images.

Matrix decomposition methods have been widely applied. Kokoya et al. [18] introduced the Coupled Non-negative Matrix Factorization (CNMF) method for unmixing, while Dian et al. [39] proposed a Non-Local Sparse Tensor Factorization (NLSTF) method. These developments highlight the evolution and diversification of techniques in the field of remote sensing data fusion.

In recent years, the advantages and advancements of deep learning in processing large datasets have increasingly gained attention in remote sensing image processing, particularly in image fusion, with a focus on convolutional approaches. Palsson et al. [40] introduced an image fusion algorithm based on 3D convolutional networks; Yang et al. [41] proposed a dual-branch fusion algorithm for extracting the spectral features of hyperspectral images and spatial domain features of multispectral images; Han et al. [42] developed a partially densely connected SSF-CNN fusion network; Dian et al. [43] introduced a fusion network based on two imaging model initializations; and Han et al. [44] introduced a multi-scale CNN method that preserves both spectral and spatial paths, progressively increasing the feature size of LR-HS and LR-MS images.

These developments show extensive research in fusion algorithms. Since the 2018 launch of GF-5, the adaptability of fusion algorithms has drawn widespread attention in the past two years. The highest spatial resolution of Sentinel-2A data is 10 m, offering a fusion ratio greater than 1:4 with GF-5 [45,46]. Therefore, this paper selects GF-5 and Sentinel-2A to explore the adaptability of fusion algorithms, choosing one representative method from each of the five categories for experimentation. The analysis of the impact of fusion algorithms on lithologic classification accuracy provides a technical reference for remote sensing lithologic classification in similar high-altitude areas.

## 2. Geological Background of the Study Area

The study area is situated in the southern reaches of Tuanjie Peak, located at the southwestern extremity of Hotan County within the Xinjiang Uyghur Autonomous Region of China. This region is a part of the Karakoram Mountains, bordered to the north by the Mazar Dahongliutan Narrow Valley and encompassing very high mountains within its terrain. The topography is characterized by higher elevations in the south and lower in the north, falling under the administrative domain of the Hotan region. The site, depicted in the regional map below, possesses an average elevation of 5500 m, presenting significant accessibility challenges for human exploration [46].

**Figure 2:**
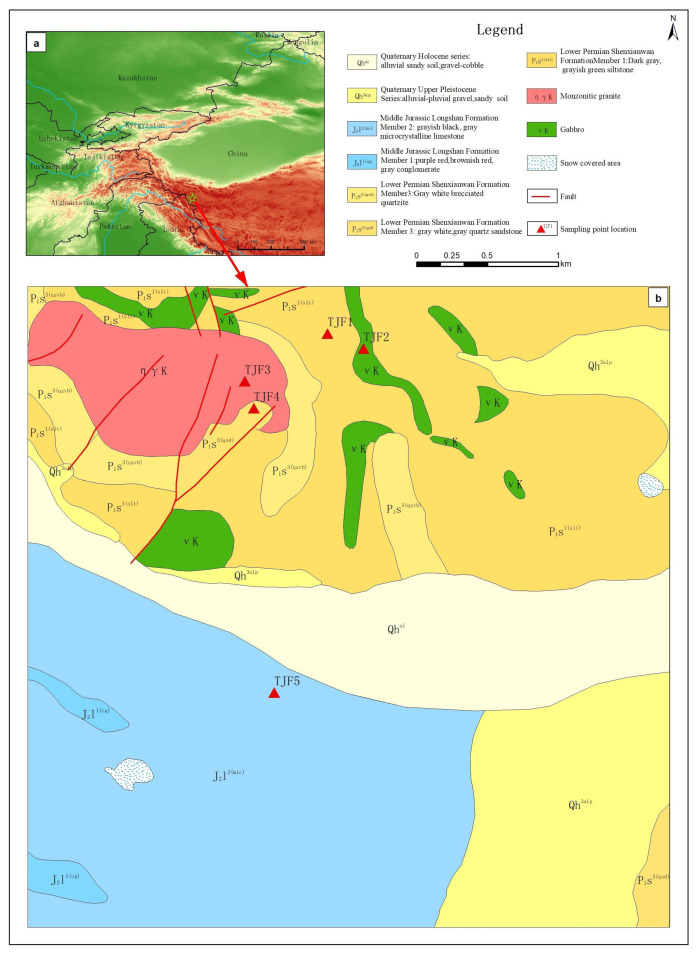
Geological Map of South Tuanjie Peak (based on a 1:50,000 geological map and remote sensing image revision, subfigure (**a**) shows the administrative location map of South Tuanjie Peak; subfigure (**b**) shows the geological sketch map of South Tuanjie Peak, which includes the location of sampling points).

Geologically, the area resides in the Kangxiwa Quanshuigou foreland basin, integral to the Qiangtang Sanjiang orogenic system. The predominant lithology of the entire southern Tuanjie Peak area comprises monzogranite, basalt, gabbro, limestone, and other strata. These are categorized into Quaternary, the Middle Jurassic Longshan Formation, and the Lower Permian Shenxianwan Formation [46]. The upper segment predominantly consists of quartz sandstone, quartz siltstone intermingled with mixed sandstone, and feldspathic quartz siltstone, interspersed locally with medium-layered ash. The Middle Jurassic Longshan Formation is differentiated into two sections—the limestone and the gravel sections [47]. In addition, there are strata from the Quaternary System, primarily consisting of the Upper Pleistocene and the Holocene series. The main lithology of the fluvial and alluvial deposits during this period is predominantly gravels and sandy soils, as illustrated in the regional geological map [48]. The 1:50,000 geological map is shown in Figure 2.

## 3. Data and Methods

This research utilizes data acquired from the GF-5 and Sentinel-2A, supplemented by the ASTGTMV003 Digital Elevation Model (DEM) data. This combination of sources provides a comprehensive dataset, encompassing high-resolution imagery and topographic information vital for the study’s objectives.

### 3.1. GF-5 and Sentinel-2A

GF-5 satellite is a full spectrum hyperspectral satellite used for comprehensive observation of the atmosphere and land in China. The satellite is equipped with two ground imaging payloads, namely the Advanced Hyper Spectral Imager (AHSI) and the Visual and Infrared Multispectral Imager (VIMI), to obtain high-resolution remote sensing data products from ultraviolet to shortwave infrared spectra. The content used from GF-5 is as shown in Table 2.

Sentinel-2 is a high-resolution multispectral imaging satellite carrying a multispectral imager (MSI) for land monitoring. It can provide images of vegetation, soil and water cover, inland waterways, and coastal areas, as well as emergency rescue services. It is divided into two satellites, 2A and 2B. The content used from Sentinel-2A is as shown in Table 3.

### 3.2. Pretreatment

The preprocessing of the GF-5 satellite data encompasses several critical steps to ensure data integrity and usability.

Following this, we introduce several key operational steps undertaken for the GF-5 satellite data, emphasizing the importance of each process in enhancing the overall quality and reliability of the hyperspectral imagery. These steps include the following:

(1) Bad Band Removal: This process involves eliminating bands impacted by water vapor absorption, specifically within the spectral wavelength ranges of 1356–1447 nm, 1800–1982 nm, and 2370–2395 nm. This is essential for accurately obtaining ground information from hyperspectral data, resulting in the utilization of 295 actual frequency bands.
(2) Radiometric Calibration: This step converts the quantified output values into radiance values (DN values). In the ENVI 5.6 software, the scale factor is set to 0.1, and the calibration coefficient for GF-5 is retrieved from the corresponding file. The output is formatted in BIL, which is crucial for subsequent atmospheric correction analysis.
(3) Bad Line Repair and Stripe Removal: Some bands of the image contain abnormal columns, where the digital number (DN) values are either 0 or significantly lower than those of adjacent pixels. These pixels are known as bad pixels, and the abnormal columns formed by them are called bad lines. The method used for bad line repair in this instance involves replacing them with the average values of adjacent columns on either side of the bad line. Vertical striping is another type of pixel value anomaly, different from bad lines. Unlike bad lines, which are single-column data anomalies, vertical striping is a kind of vertical band anomaly with smaller DN values that differ markedly from the surrounding environment. The local destriping method is used to correct vertical striping.
(4) Atmospheric Correction: This study mainly used the FIAASH atmospheric module in ENVI software for atmospheric correction. The FIAASH atmospheric module within ENVI software is primarily used for this purpose, correcting for atmospheric distortions in the imagery. The main parameters for atmospheric correction are as shown in Table 4.
(5) MNF Noise Separation: In ENVI, this module is utilized to determine image dimensions, isolate noise, and minimize processing and computational demands. The process involves dividing into high-resolution bands in the visible–near-infrared (VNIR) and shortwave infrared (SWIR) spectra, retaining the first 20 bands for inverse MNF rotation, and subsequently stacking to obtain bands of hyperspectral images.
(6) Orthorectification: To eliminate geometric distortions caused by terrain and other factors, orthorectification of GF-5 imagery is conducted on the ENVI 5.6 platform using the inherent RPC parameters of GF-5 data, along with concurrent Landsat 8 imagery data and ASTGTMV003 30m elevation data. This process ensures that the error with the reference image is controlled within one pixel, providing support for accurate subsequent fusion and classification.

The Level 1A-C product of the Sentinel-2A satellite is meticulously calibrated for both radiometric and atmospheric corrections using advanced sensors provided by the European Space Agency (ESA). Subsequently, these calibrated data are standardized using the Sentinel Application Platform (SNAP) 8.0 software, ensuring uniformity and consistency for further analysis.

### 3.3. Data Fusion Methods

This article primarily employs five methods for fusion research: Gram-Schmidt Adaptive (GSA) [14], Smoothing-Filter based Intensity Modulation(SFIM) [49], Coupled Non-negative Matrix Factorization (CNMF) [50], Hyperspectral Image Super Resolution Method (Hysure) [51], and a Non-rigid Registration Hyperspectral Super-Resolution Network (NonRegSRNet) [52].

### 3.4. Fusion Effect Evaluation Method

These metrics are Peak Signal-to-Noise Ratio (PSNR), Spectral Angle Mapper (SAM) [53], Relative dimensionless global error in synthesis (ERGAS) [54], $Q2^{n}$ [55], $D_{\lambda }$, $D_{s}$, Quality with No Reference (QNR) [56], and AG.

#### 3.4.1. Peak Signal-to-Noise Ratio, PSNR

The peak signal-to-noise ratio (PSNR) is a very popular quality metric, for two grayscaling images A and B,

(1) $$\mathrm{PSNR}\left(A,B\right)=20\log\frac{255}{\mathrm{RMSE}(A,B)}$$

in which the root-mean-square error (RMSE) is expressed as

(2) $$\mathrm{RMSE}\left(A,B\right)=\sqrt{\frac{||A-{B||}_{F}^{2}}{N}}$$

in which N represents the number of pixels. The PSNR for HSI is defined as average value of all bands. A bigger value of the PSNR means better fusion results.

#### 3.4.2. Spectral Angle Mapper, SAM

The SAM is a very crucial index to evaluate the spectral distortions and is defined as

(3) $$\mathrm{SAM}\left(X,\hat{X}\right)=\frac{1}{M}\sum_{j=1}^{M}\arccos\frac{{\hat{X}}^{j}\cdot X^{j}}{\left|\right|X^{j}{\left|\right|}_{2}\left|\right|{\hat{X}}^{j}{\left|\right|}_{2}}$$

in which *M* is the number of spectral pixels, and · denotes inner product of two vectors. A smaller value of SAM means fewer spectral distortions.

#### 3.4.3. Relative Dimensionless Global Error in Synthesis, ERGAS

The relative dimensionless global error in synthesis (ERGAS) is represented as follows:

(4) $$\mathrm{ERGAS}\left(X,\hat{X}\right)=\frac{100}{d}\sqrt{\frac{1}{S}\sum_{i=1}^{S}{\left(\frac{\mathrm{RMSE}(X_{i},\hat{X_{i}})}{\mu (\hat{X_{i}})}\right)}^{2}}$$

in which *d* is spatially sub-sampling factor, and μ represents the mean value of the image. The smaller ERGAS, the better the fusion results.

#### 3.4.4. $Q2^{n}$

$Q2^{n}$ is a generalization of the universal image quality index (UIQI) and an extension of the Q4 index to HS images based on hypercomplex numbers. The UIQI or the *Q* index was proposed by Wang and Bovik to measure any distortion in monochromatic images as the product of three factors, i.e., loss of correlation, luminance distortion, and contrast distortion. The UIQI between one reference image band (*x*) and its corresponding target image band (*y*) is defined as follows:

(5) $$Q\left(x,y\right)=\frac{4\sigma_{xy}\bar{x\gamma }}{\left(\sigma_{x}^{2}+\sigma_{y}^{2}\right){\left({\bar{x}}^{2}+{\bar{\gamma }}^{2}\right)}^{\prime }}$$

Although this coefficient can be complex in calculation, it encapsulates a comprehensive range of physical information. Its value range aligns with that of UIQI, typically residing within the (0, 1) interval. A value approaching 1 indicates superior spatial quality of the fusion, as well as enhanced correlation with the reference image.

#### 3.4.5. $D_{\lambda }$, $D_{S}$ and QNR

For fusion metrics of images without a reference, commonly used are the $D_{\lambda }$ (spectral component of QNR), the $D_{S}$ (spatial component of QNR), and QNR (quality with no reference). $D_{\lambda }$ assesses the spectral quality of the fused image, $D_{S}$ evaluates the quality of spatial restoration in the fused image, and QNR serves as an index for assessing the overall quality of the fused image. The specific formulas are as follows:

(6) $$D_{\lambda }≜\sqrt[{p}]{\frac{1}{L(L-1)}\sum_{I=1}^{L}\sum_{\begin{array}{c} r=1\\ r\neq 1 \end{array}}^{L}{\left|Q\left({\hat{G}}_{I},{\hat{G}}_{r}\right)-Q\left({\tilde{G}}_{I},{\tilde{G}}_{r}\right)\right|}^{P}}$$

In the above formula, ${\hat{G}}_{I}$ represents the upsampled hyperspectral image, ${\tilde{G}}_{I}$ is the fused image, *L* denotes the number of bands, and *Q* represents the degree of band distinctiveness. The most ideal value for $D_{\lambda }$ is 0.

(7) $$D_{s}≜\sqrt[{q}]{\frac{1}{L}\sum_{I=1}^{L}{\left|Q\left({\hat{G}}_{I},P\right)-Q\left({\tilde{G}}_{I},\tilde{P}\right)\right|}^{q}}$$

In the given equation, *P* refers to the multispectral (MS) image, which undergoes spatial degradation subsequent to low-pass filtering. The optimal value for $D_{s}$, indicative of this degradation, is 0.

(8) $$QNR≜{\left(1-D_{\lambda }\right)}^{\alpha }\cdot {\left(1-D_{s}\right)}^{\beta }$$

The equation incorporates α, β which serve as weighting parameters. For these parameters, an ideal metric value is established at 1, signifying optimal weighting in the fusion process.

#### 3.4.6. Average Gradient, AG

The Average Gradient (AG) index serves as a metric for assessing the clarity of a fused image. The underlying principle is that a higher average gradient correlates with increased detail and, consequently, improved image clarity and fusion quality. In this context, *A* and *B* denote the width and height of the image, respectively, while I represents the pixel value at the (i,j) position within the image.

(9) $$AG\left(X\right)=\frac{1}{\left(A-1)(B-1\right)}\sum_{i=1}^{A-1}\sum_{j=1}^{B-1}\sqrt{\frac{\left({\left(I\left(i+1,j\right)-I\left(i,j\right)\right)}^{2}+{\left(I\left(i,j+1\right)-I\left(i,j\right)\right)}^{2}\right)}{2}}$$

### 3.5. Image Classification Methods and Evaluation Metrics

The Random Forest (RF) algorithm, a nonlinear and nonparametric classifier, was initially introduced by Breiman et al. in 2001 [57]. It is an evolution of the Bagging algorithm and is adept at employing multiple trees for the classification, training, and prediction of datasets. This makes it particularly effective for categorizing high-dimensional and complex data, offering outstanding robustness and generalizability.

The overall accuracy is calculated as the ratio of the sum of correctly classified pixels to the total pixel count. The accuracy of surface classification is determined by the actual surface imagery or defined areas of interest. In this study, the accurate distribution of each lithological type is ascertained by totaling the pixels corresponding to each rock type, thereby determining the classification accuracy for each type.

The Kappa Coefficient is a quantitative measure used to evaluate the consistency or accuracy Coefficient as follows:

(10) $$K_{hat}=\frac{N\sum_{i=1}^{n}X_{ij}-\sum_{i=1}^{n}\left(X_{i+}X_{+i}\right)}{N^{2}-\sum_{i=1}^{n}\left(X_{i+}X_{+i}\right)}$$

In this equation, *n* represents the total number of columns in the confusion matrix, which corresponds to the total number of categories. $X_{ij}$ denotes the count of pixels located at the intersection of the ith row and jth column within the confusion matrix, signifying the quantity of correctly classified pixels. $X_{i+}$ and $X_{+i}$ are the sums of the pixels in the ith row and ith column, respectively. *n* is the aggregate number of pixels utilized in the evaluation process.

## 4. Experimental Design, Analysis, and Discussion

### 4.1. Experiment and Results

After data preprocessing, all 295 bands of GF-5 are 30 m, whereas Sentinel-2A retains four 10 m bands, 2, 3, 4, and 8, for fusion experiments. The central wavelength of the B2 band is 490 nm, B3 band is 560 nm, B4 band is 665 nm, and B8 band is 842 nm.

Given the lower clarity of GF-5, conducting fusion experiments with GF-5 can enhance its spatial resolution and facilitate subsequent lithology classification.

Considering the extensive data volume of the study area, a lithologically diverse region was chosen for the fusion experiments.

**Figure 3:**
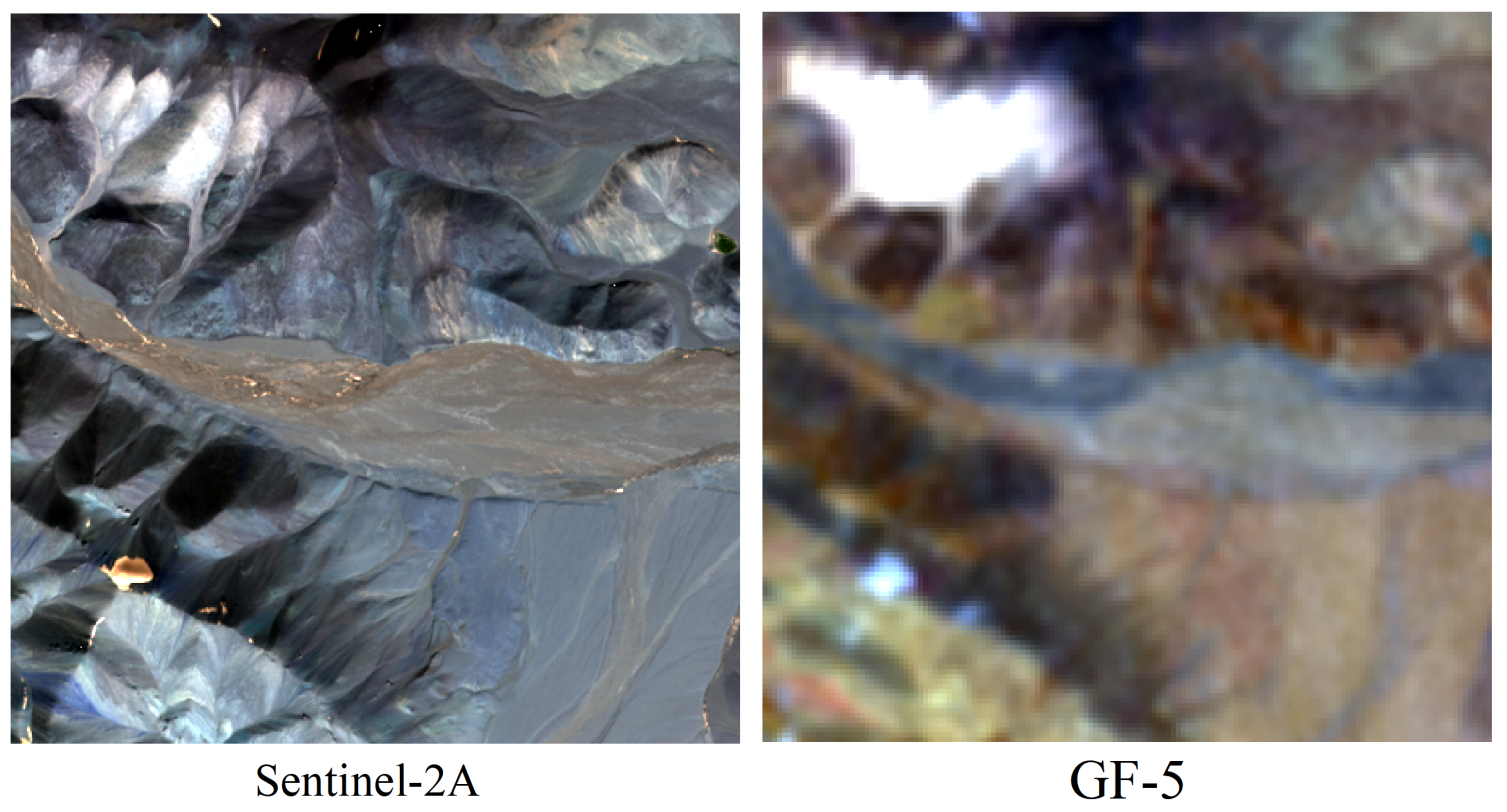
Original image data.

For this purpose, GF-5 images from the southern region of Tuanjie Peak were segmented into 150 × 150-pixel sub-images, while Sentinel-2 images were divided into 450 × 450-pixel sub-images. The remote sensing images of the two datasets are shown in Figure 3.

The fusion algorithm experiments based on GSA, SFIM, CNMF, and HySure are conducted in Matlab2022a software, using these algorithms to read and fuse sub-images of GF-5 and Sentinel-2A. For the fusion algorithm based on NonRegSRNet, the prepared sub-images are used as test data, while the remaining areas are used for training. There is no overlap between the test and training areas. In total, 80% of the data are used for training and 20% for testing. The experiments are implemented using the Python 3.7 language on the PyTorch framework.

To account for spectral characteristic discrepancies in multi-source imagery of identical land objects, relative radiometric normalization and Spectral Response Function (SRF) computation were prerequisite steps in the NonRegSRNet fusion algorithm. Table 5 details the parameters, defaulting to the method’s inherent settings except where noted.

Training loss curves, visualized using the PyTorch tool visdom, typically stabilized between 300 to 400 training iterations, facilitating an effective observation of loss trends.

**Figure 4:**
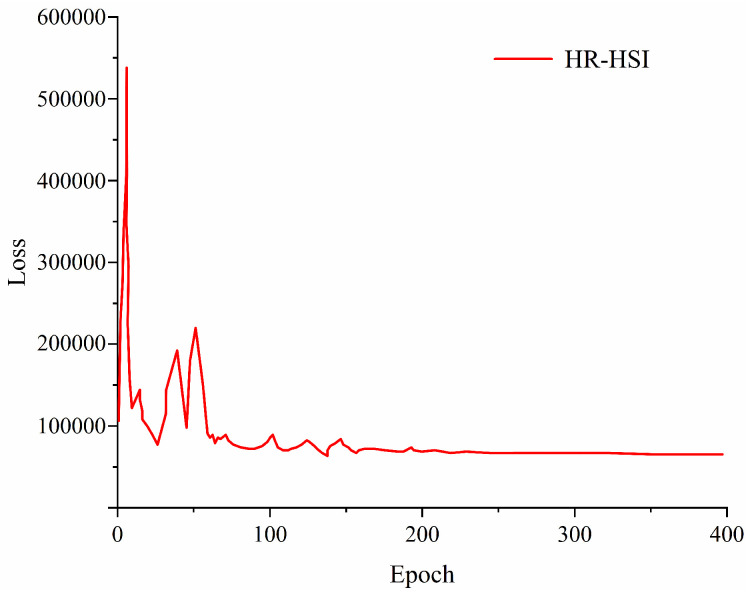
Fusion process loss curve.

The fusion outcomes employing GSA, SFIM, CNMF, Hysure, and NonRegSRNet are depicted in Figure 4.

#### 4.1.1. Visual Evaluation

**Figure 5:**
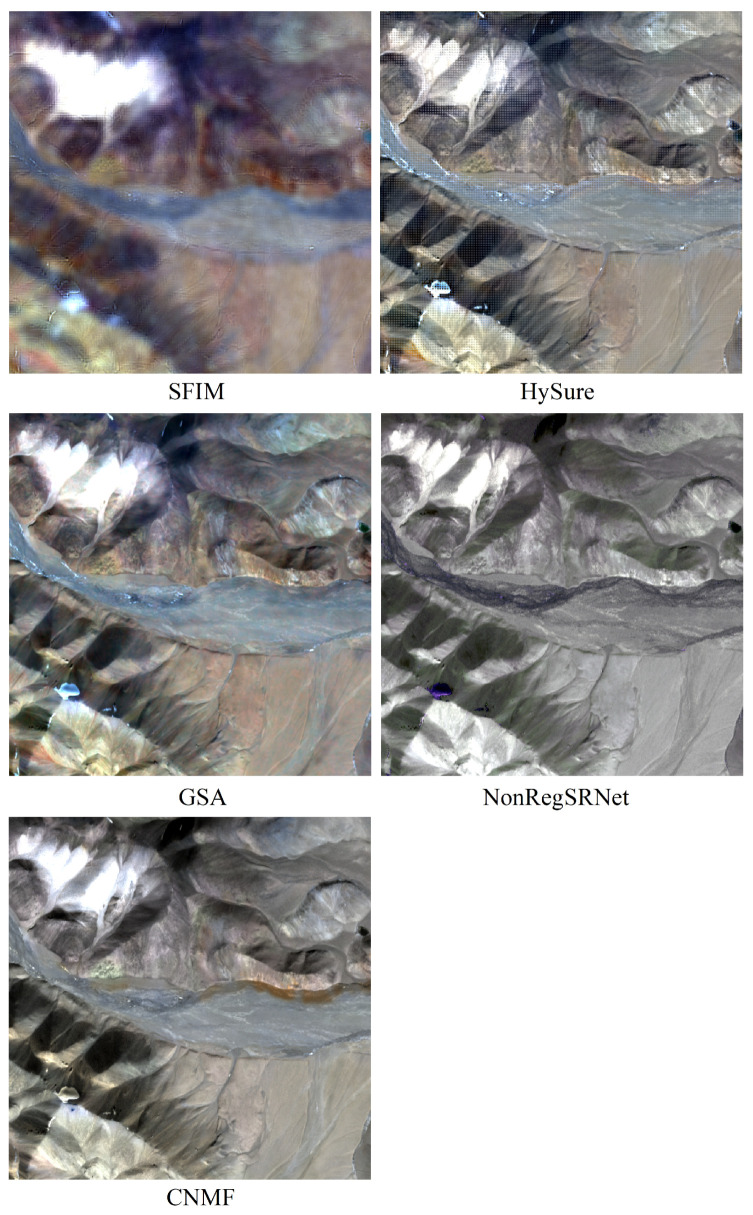
Remote sensing image fusion result.

As observed in Figure 5, the SFIM technique fails to significantly enhance spatial resolution, resulting in relatively blurred fusion outcomes where lithological and geological features are not distinctly portrayed. In contrast, GSA, HySure, and CNMF methods markedly improve the spatial resolution, rendering clearer lithological textures, albeit with minor variations in color fidelity. NonRegSRNet, however, produces monochromatic land object representations, lacking in accurately depicting lithological variations and characteristics.

Consequently, GSA, HySure, and CNMF demonstrate superior performance in terms of visual clarity in lithology, whereas SFIM shows subpar clarity, and NonRegSRNet exhibits limited color diversity. To delve deeper into these variances, subsequent steps will involve the application of quantitative analysis using specific evaluation metrics.

#### 4.1.2. Indicator Assessment

Firstly, this arcticle uses eight indices for comprehensive evaluation, PSNR, SAM, ERGAS, $Q2^{n}$, $D_{\lambda }$ (spatial distortion), $D_{s}$ (spectral distortion), QNR (no-reference image evaluation index) and AG (average gradient), with the results shown in Table 6. We analyzed based on the table results as follows:

The evaluation of the image quality post-fusion is carried out using these eight indices, discussing from both referenced and non-referenced aspects. PSNR, SAM, ERGAS, and $Q2^{n}$ are referenced image evaluation indices (using the original hyperspectral image as the reference); $D_{\lambda }$, $D_{s}$, QNR, and AG are non-referenced image evaluation indices, being determined by $D_{\lambda }$ and $D_{s}$.

In the PSNR index, GSA fusion achieved the highest value, indicating the best spatial reconstruction. GSA showed the lowest in the SAM index, suggesting the best spectral quality. In the ERGAS index, NonRegSRNet was the smallest, implying the best global statistical measure of fused data quality. GSA was closest to 1 in the $Q2^{n}$ index, indicating good spectral fidelity and minimal spatial distortion. In the $D_{\lambda }$ index, NonRegSRNet was closest to 0, denoting it as the optimal choice; similarly, NonRegSRNet was nearest to 0 in the $D_{s}$ index, also indicating that it is the best. As NonRegSRNet had the best results in both $D_{\lambda }$ and $D_{s}$, it was also the best in the QNR index. GSA performed best in the AG index, showing the highest clarity.

**Figure 6:**
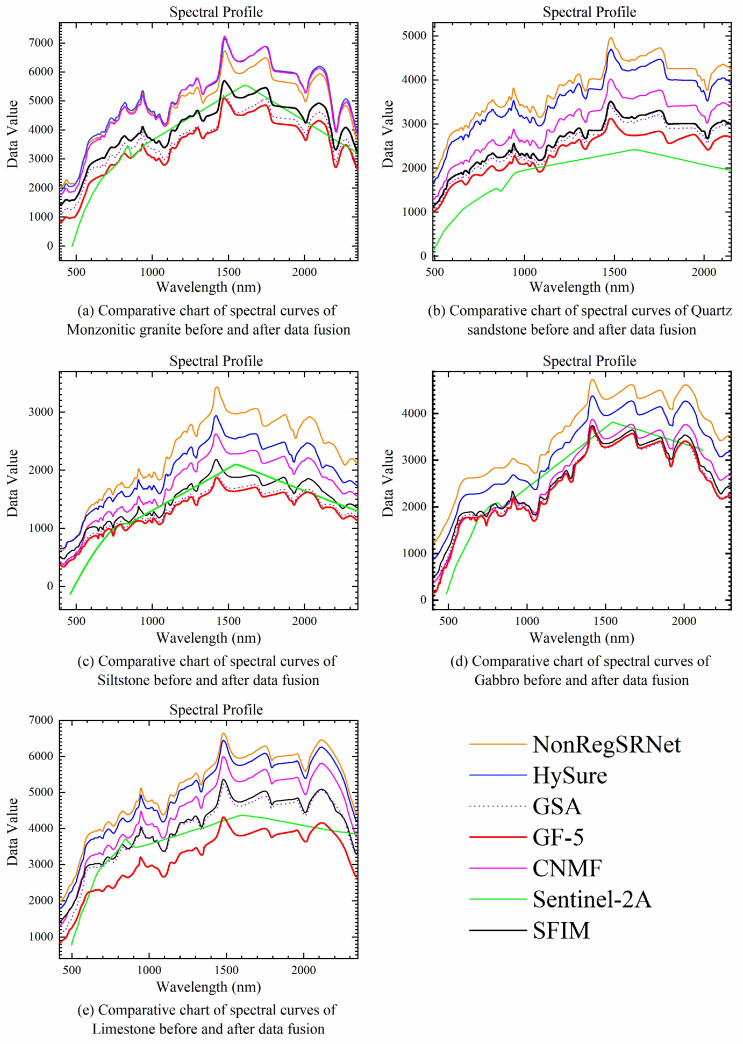
Spectral curves of five typical rocks before and after data fusion.

Secondly, we compared the spectra of the main lithologies and features in the study area before and after fusion, with the results shown in Figure 6. From Figure 6, it can be seen that the spectral curves of the five main lithologies in the remote sensing images fused by GSA are closest to the pre-fusion spectral curves. The spectral distortions in the NonRegSRNet and HySure fused spectra mainly manifest as slight increases in reflectance, but the overall spectral shape remains fairly consistent with the original spectra. This is in line with the conclusion from the SAM index evaluation that GSA has the best spectral quality. Therefore, a comprehensive analysis of the indices and spectral analysis shows that GSA has the best fusion effect in this experiment.

#### 4.1.3. Evaluation of Lithology Classification Performance

**Figure 7:**
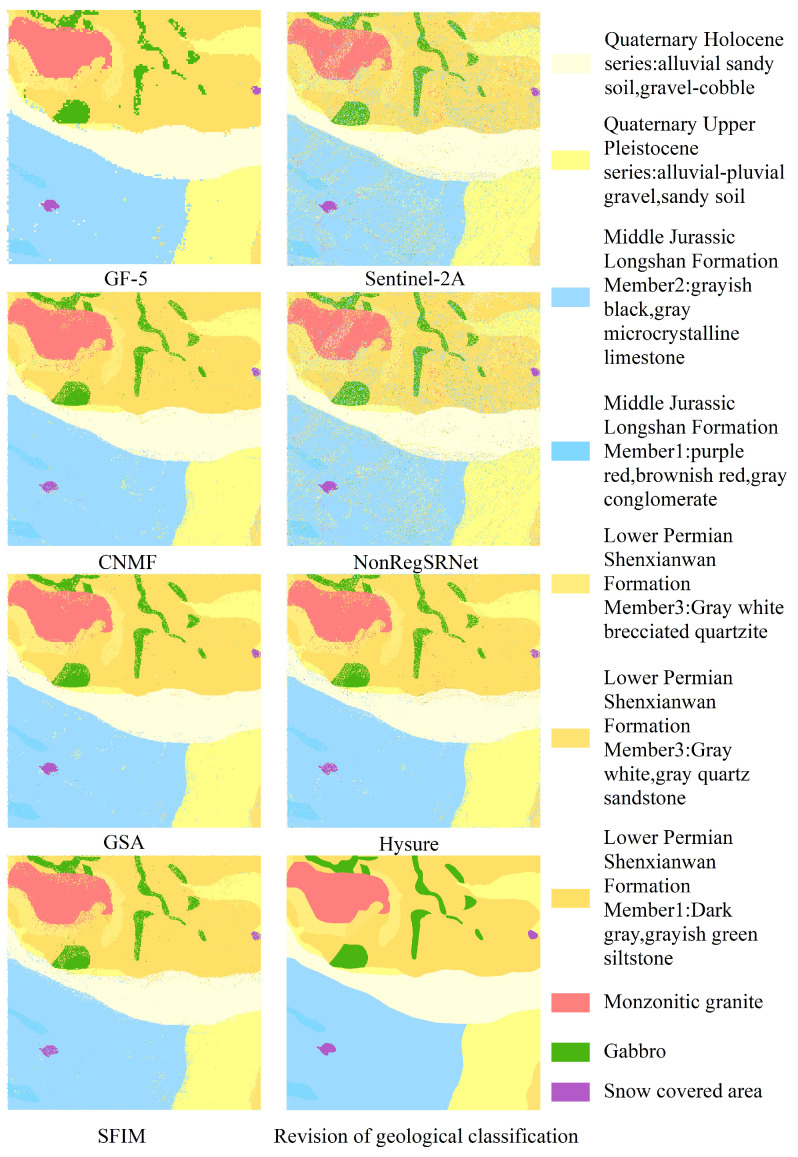
Area sample classification results.

**Figure 8:**
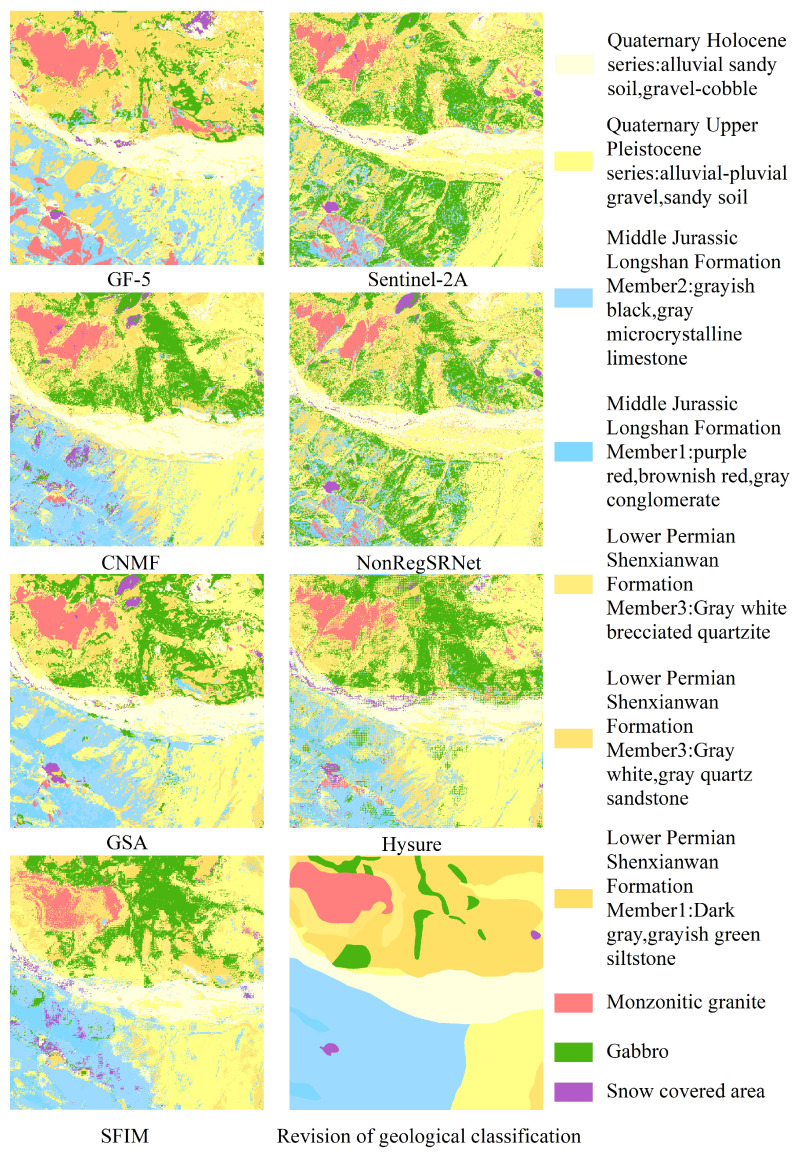
Point sample classification results.

In the current research, the Random Forest (RF) classification methodology was employed to compare lithological classifications between individual and fused images. The RF classification process was executed using the sklearn library [58]. For parameter optimization, a grid search approach was adopted, setting n_estimators to 120 and max_depth to 2, while maintaining default settings for all other parameters. Aiming to enhance the accuracy of lithology classification, we selected samples for analysis through both point-based and area-based approaches. The distribution of training rock samples and test samples is depicted in Table 7. Python programming was utilized to automatically allocate these samples into a 70:30 training-to-testing ratio. The classification was then applied to pre-fusion GF-5 and Sentinel-2 images, as well as images resulting from five different fusion methods, with the outcomes presented in Figure 7 and Figure 8.

In this study, our initial focus was on the delineated imagery presented in Figure 7 and Figure 8. We noted that the periphery of each classified category in the surface sample training results predominantly aligns well. However, some categories included noise points and a mixture of other classification types. The performance of point sample training was moderate, with notable misclassification in certain categories.

**Figure 9:**
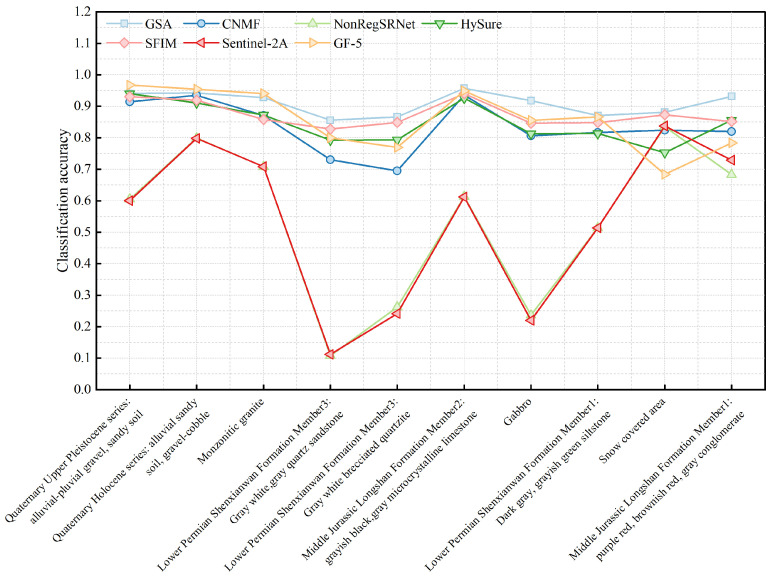
Accuracy charts for each category of area samples.

**Figure 10:**
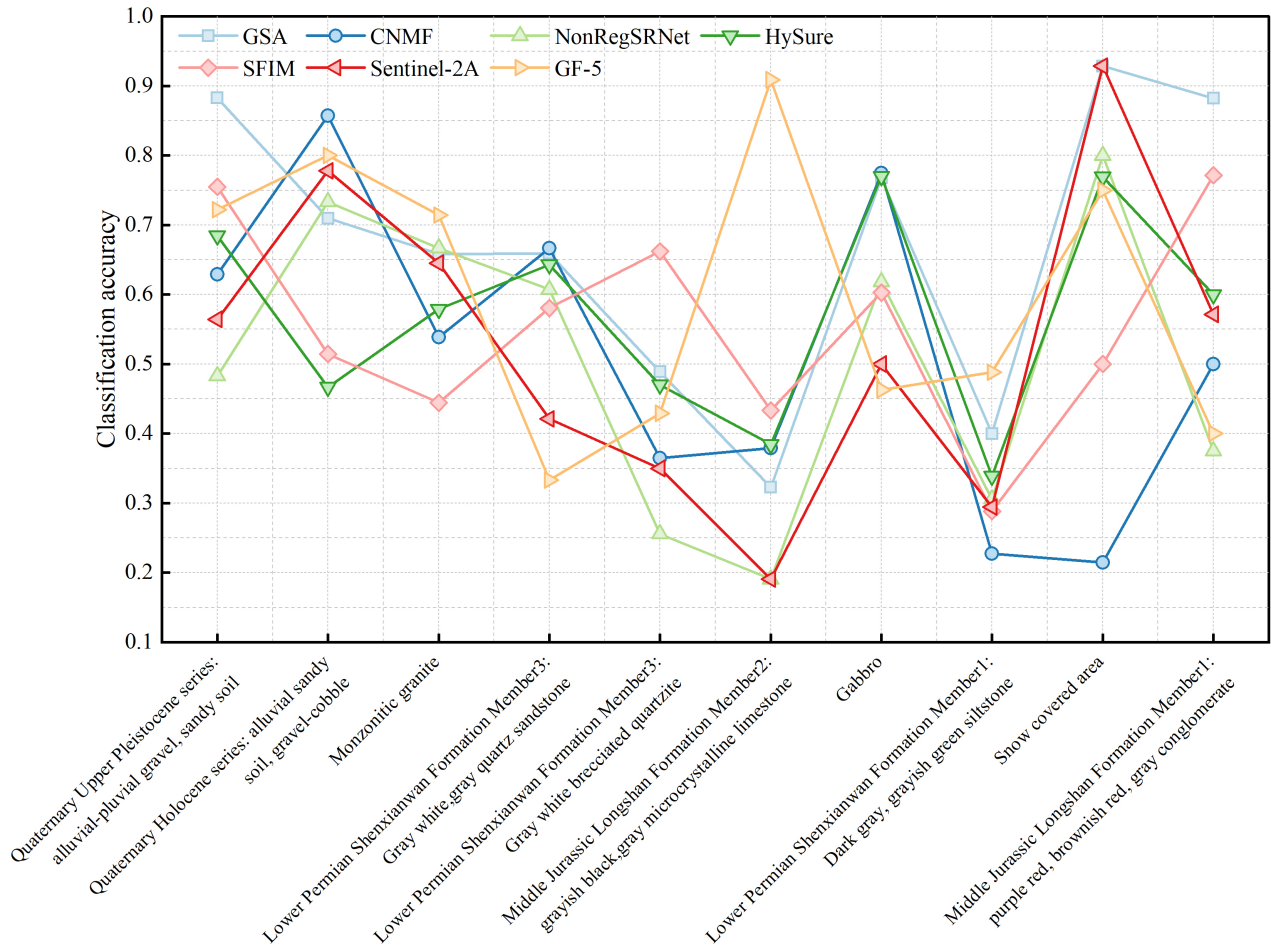
Accuracy charts for each category of point samples.

The examination of Figure 9 and Figure 10 indicated a general balance among the categories within the surface samples. Apart from the exclusive use of Sentinel-2A data and the NonRegSRNet fusion method, the accuracy across the categories was commendably high. In contrast, point samples demonstrated considerable variability in accuracy, with a tendency towards lower precision compared to surface samples.

**Figure 11:**
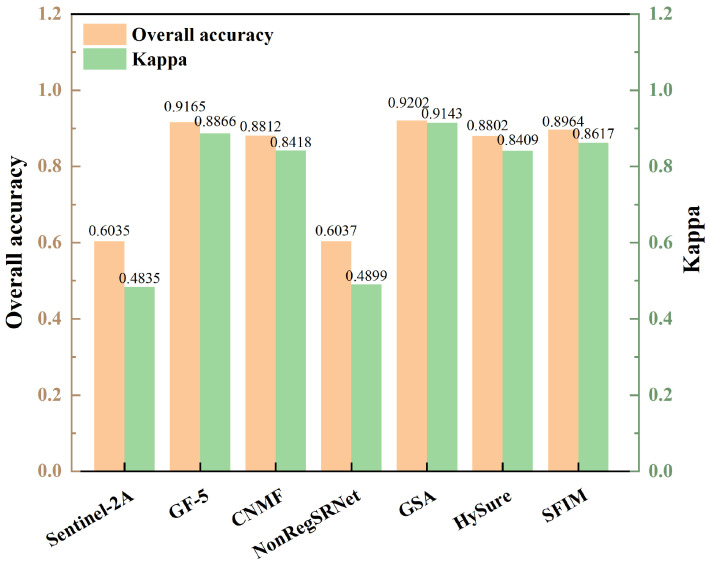
Overall classification accuracy and kappa coefficient for area samples.

**Figure 12:**
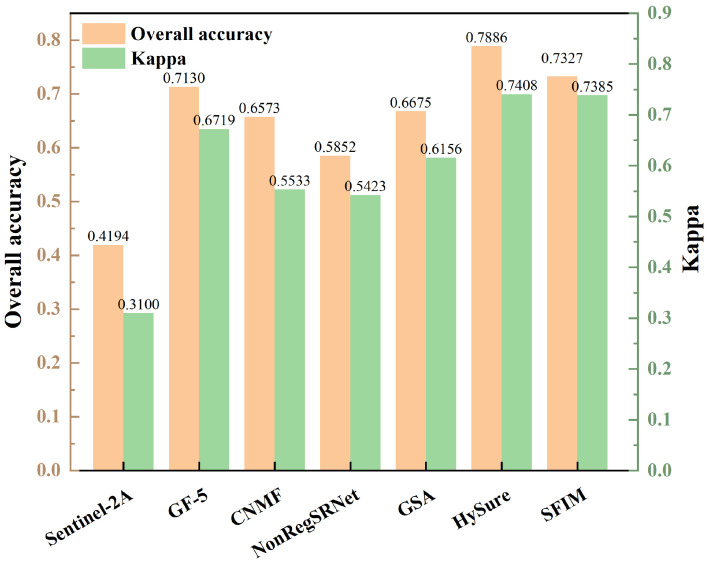
Overall classification accuracy and kappa coefficient for point sample.

An analysis of the overall classification accuracy and kappa coefficient, as shown in Figure 11 and Figure 12, was crucial for a nuanced understanding of the differences between surface and point samples. This paper, therefore, undertakes a detailed discussion and analysis of both sample types.

Evaluating the classification accuracy of individual lithologies, we observed that surface samples maintained a relatively stable accuracy across categories. This was with the notable exception of those utilizing Sentinel-2A data and the NonRegSRNet fusion method. Point samples, however, showed more pronounced fluctuations in accuracy, underperforming compared to their surface counterparts. It was clear that for surface samples, each lithology category achieved exceptional results with the GSA fusion method. Specifically, the Jurassic Longshan Formation’s second section, characterized by grey-black and grey microcrystalline limestone $\left(J_{2}I^{2(mic)}\right)$, exhibited superior performance with the GSA fusion method. While the GF-5 data showed some discrepancies in lithology classification, the Quaternary Holocene $\left(Qh^{3alp}\right)$ stood out as the bestperforming category. In point samples, the same section of the Jurassic Longshan Formation $\left(J_{2}I^{2(mic)}\right)$ excelled when using GF-5 data, with the Quaternary Holocene $\left(Qh^{3alp}\right)$ achieving the highest accuracy with the GSA fusion method. This analysis suggests that the recognition accuracy of $J_{2}I^{2(mic)}$ and $Qh^{3alp}$ is commendably high in both point and surface samples within the studied region.

In evaluating the classification accuracy of individual lithologies through various fusion methods, our analysis revealed that surface samples using the GSA fusion method, standalone GF-5 data, and SFIM fusion exhibit a commendable performance. The majority of lithologies in these samples surpass the average user accuracy. For point samples, the GSA and CNMF fusion methods stand out, delivering superior results, whereas the sole use of Sentinel-2A data is markedly less effective, showing substantial variability in lithology classification across different datasets.

Upon examining the data sources, it is evident that GSA-based fusion data consistently offers the most accurate lithology classifications. The un-fused GF-5 data rank second, outperforming the un-fused Sentinel-2A data, which show weaker results across all lithologies. Notably, the lithology classifications from GF-5 data, especially for the Quaternary Holocene $Qh^{3alp}$, biotite granite ηγ*K*, the middle Jurassic Longshan Formation’s second section $J_{2}I^{2(mic)}$, and the lower Permian Shenxianwan Formation’s first section $P_{1}s^{3(s1t)}$, are more precise than certain fused datasets. This could be attributed to spectral distortions resulting from the fusion process. For instance, the NonRegSRNet fusion method, while scoring well on some fusion evaluation metrics, suffers from inadequate spatial reconstruction and significant spectral distortion, leading to reduced classification accuracy of individual lithologies. Similarly, the SFIM fusion method, despite its initial promise, results in images of inferior spectral and spatial quality, ultimately compromising the classification accuracy compared to the pre-fusion GF-5 data. The overall lower accuracy of Sentinel-2A data in lithology classification further underscores that high spatial resolution alone is inadequate for accurate lithology classification in this region. A high spectral resolution is essential to meet the classification requirements effectively.

In this research, lithology classification outcomes were meticulously examined through two lenses, lithology classification accuracy and the kappa coefficient, as detailed in Table 8 and Figure 9. A closer look at the overall lithology classification accuracy in Figure 11 and Figure 12 reveals a consistent trend with the single lithology classification accuracy. Notably, the GSA method in surface samples attained the apex of overall lithology classification accuracy at 92.02%, coupled with a robust kappa coefficient of 0.9143. Meanwhile, the HySure fusion method shone in point samples, achieving the highest overall classification accuracy of 78.86%. It is important to highlight that across both point and surface samples, the classification accuracy of the single Sentinel-2A data consistently lagged, ranking as the lowest among the fusion data.

Furthermore, Figure 11 unveils that the un-fused remote sensing image GF-5’s overall lithology classification accuracy and kappa coefficient were marginally surpassed by GSA. However, the performance metrics for Sentinel-2A were distinctly at the lower end. This analysis underscores the superiority of GF-5 in lithology classification, primarily attributed to its higher spectral resolution. Despite the high spatial resolution of the single Sentinel-2A data, its lower spectral resolution proved to be a limiting factor in effective lithology recognition.

### 4.2. Field Verification

#### 4.2.1. Field Verification and Microscopic Verification

**Figure 13:**
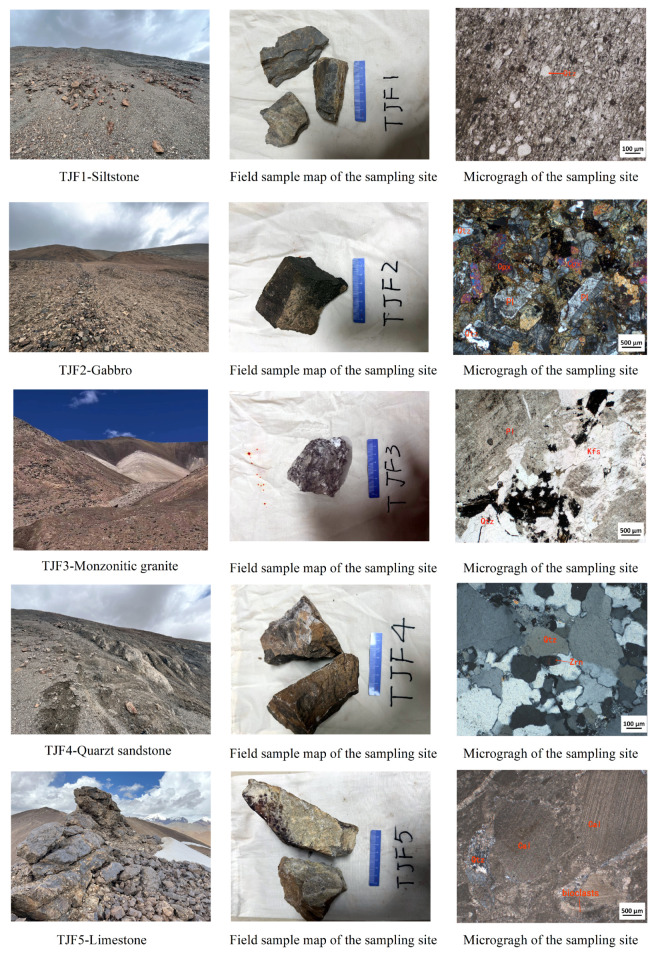
Images related to three field sampling locations.

In this study, our selected research area is situated in a challenging high-altitude region, complicating the process of field verification. To address this, we strategically chose five distinct field sampling points. The first point, known as TJF-1, is located within the lower section of the Permian Shenxianwan Formation and primarily consists of siltstone. The second point, TJF-2, is situated in the diorite stratum. TJF-3, the third point, is found within the Monzonitic granite. The fourth point, TJF-4, lies in the third section of the lower Permian Shenxianwan Formation, characterized by quartz sandstone. The fifth and final point, TJF-5, is part of the middle Jurassic Longshan Formation’s second section, identified by its limestone composition. A comprehensive view of these field sampling locations, including panoramic photographs, hand specimen images, and detailed microscopic photographs, is meticulously documented in Figure 13.

Through field validation and microscopic identification of five types of lithology samples, and using 1:50,000 geological maps as a reference, the consistency between lithology and classification results demonstrates the accuracy of the fusion experiment.

#### 4.2.2. Image and Actual Spectral Verification

**Figure 14:**
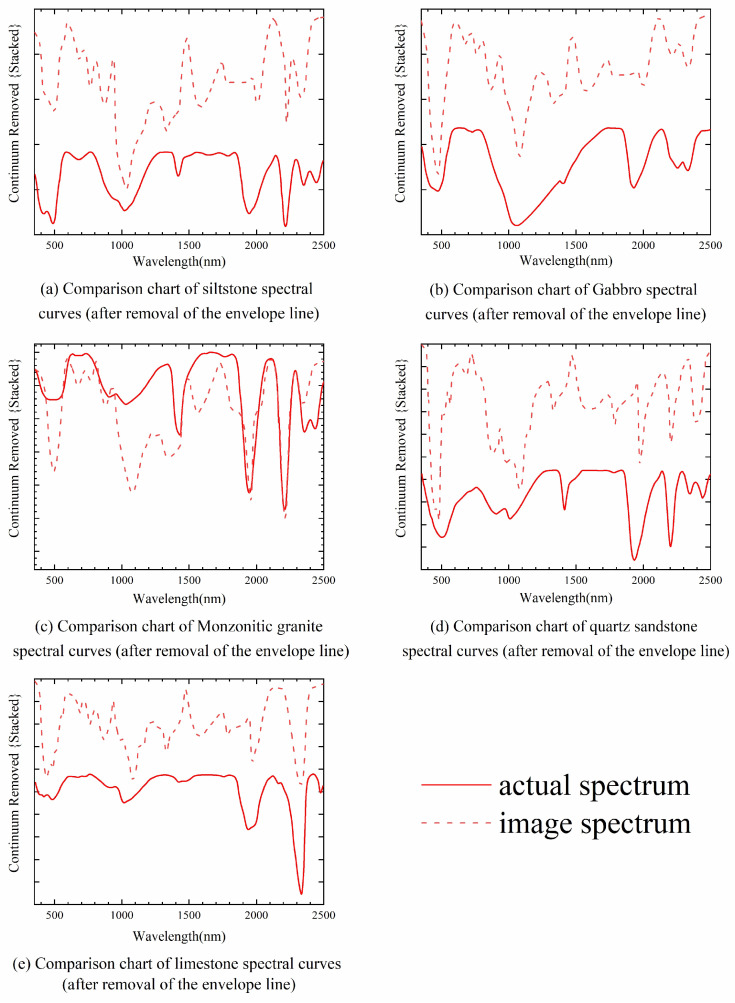
Comparison between actual spectral curves and image spectral curves.

We conducted indoor spectral measurements on the collected rock samples, repeatedly collecting them from both weathered and fresh surfaces. We took the average of each spectral measurement to represent the spectral signature of the lithological samples. After processing, we exported the spectral curves of the lithological samples at the sampling points. To more clearly discern the lithological characteristics, we compared the rock sample spectral curves with the image spectral curves after removing the envelope line. The comparative diagram is shown in Figure 14.

Through meticulous field validation efforts, we have established a clear alignment between the observed lithology and the classification outcomes. This correlation not only validates the accuracy of the experimental procedures undertaken but also highlights the significant potential of remote sensing technology in broader applications. This achievement not only reinforces the reliability of our experimental findings but also paves the way for the wider utility and application value of remote sensing methodologies in geological studies and beyond.

Figure 14a shows the spectrum of siltstone. Microscopic identification reveals its structure as silty, accompanied by altered structures, and blocky in formation. The main mineral components are quartz and plagioclase, with minor amounts of hornblende. The secondary minerals mainly include sericite, soapstone, and green earth, among others. Quartz itself lacks diagnostic features in visible and shortwave infrared light, but the presence of sericite in siltstone, which contains Al-OH spectral features, results in absorption features around 2200 nm and 1900 nm. The greater absorption depth at 2200 nm confirms the accuracy of the field validation of the siltstone.

Figure 14b shows the spectrum of Gabbro. Microscopic identification reveals its structure as fine-grained diorite, with a blocky formation. The main mineral components are plagioclase, followed by monoclinal pyroxene, with minor amounts of hornblende, titanium iron oxides, and quartz. The secondary minerals mainly include sericite, soapstone, and green earth, among others. Therefore, the image also shows the spectral absorption characteristics of Al-OH and the dual absorption features of Mg-OH minerals around 2200 nm and 2300 nm. This confirms the accuracy of the field identification of Gabbro.

Figure 14c shows the spectrum of monzonitic granite. Microscopic identification reveals its structure as granite porphyry, with a blocky formation. The main mineral components in the rock are plagioclase, potassium feldspar, and quartz, with minor amounts of biotite, muscovite, and black opaque minerals. Therefore, absorption features also appear at 1900 nm and 2200 nm, confirming the accuracy of the field validation of the monzonitic granite.

Figure 14d shows the spectrum of quartz sandstone lithology. Under microscopic examination, its structure is identified as a medium to fine-grained sandy structure with a massive texture. The primary mineral components in the rock are quartz, with minor amounts of feldspar and white mica. Consequently, characteristic absorption peaks at 1900 nm and 2200 nm are also observed, confirming the accuracy of field validation for quartz sandstone.

Figure 14e presents the limestone spectrum. Under microscopic analysis, its structure is identified as micro- to fine-grained granular and crystalline, with a massive texture. The primary components are quartz, calcite, and bioclasts. As calcite is a carbonate rock mineral, characteristic absorption features of ${{\mathrm{CO}}_{3}}^{2-}$ are observed near 2330 nm, confirming the accuracy of field validation for limestone.

From the combined results of field, spectral, and microscopic analyses of the five lithologies, it is evident that the validation results are satisfactory, with spectral characteristics aligning with the primary minerals of the lithologies. However, due to the high altitude of the study area, field validation is relatively challenging. Therefore, this study only conducted field and spectral validations in five accessible areas. This demonstrates the applicability of the integrated method used in this study for lithological classification in the region, yielding accurate classification results. This method can provide technical support for geological work such as lithological surveys in similar high-altitude areas.

## 5. Conclusions

This paper focuses on the study and comparative analysis of image fusion methods in selected typical areas of the Tuanjie Peak region. Five representative fusion methods were chosen, and research was conducted from three aspects, index evaluation and spectral analysis, comparison of lithology classification results, and field and spectral verification, leading to the following conclusions:

Firstly, a comprehensive index analysis and spectral analysis indicate that GSA achieves the best fusion effect in this experiment. Secondly, lithology results from RF show that, with geological maps as a reference, the classification accuracy of area samples is higher. The classification results of point samples are more dispersed, and lithological boundaries are not distinct. There may be instances of intermingling among actual lithologies like sandstone, mudstone, and conglomerate. Therefore, precise field data are required to validate the actual classification effectiveness of point samples.

Furthermore, looking at the classification results of area samples, lithology classification based on GSA shows outstanding accuracy, with each lithology’s precision higher than the average user accuracy. The classification accuracy of standalone GF-5 data is only slightly less than that of GSA. Overall, GSA fusion yields the highest classification accuracy, reaching 92.02%, with the highest kappa coefficient at 0.9143. The overall lithology classification accuracy and kappa coefficient of the unfused remote sensing image GF-5 are only slightly lower than that of GSA fusion. However, the classification accuracy and kappa coefficient of standalone Sentinel-2A are the lowest.

Finally, from the perspective of field and spectral verification, the results from field observation, microscopic examination, and spectral validation are good, reflecting the effectiveness of lithology classification based on GSA as discussed in this paper. Therefore, in the Tuanjie Peak region, the GSA fusion method is the best fusion approach for the area, capable of enhancing the lithology classification effect of standalone Gaofen data or Sentinel data. The combination of “GSA fusion+RF” can be used as a technical approach for lithology classification recognition in this area.

This paper only discusses the applicability of five fusion methods for lithology classification in the area. The poor performance of deep learning may be due to the NonRegSRNet algorithm not being suitable for fusion in this area. In the future, more deep learning fusion methods could be tried, with research focusing on image fusion, lithology identification, or other remote sensing geological information recognition. Additionally, to facilitate computation and verification in accessible areas, experiments were only conducted in a portion of the area. In the future, discussions and research on the applicability of methods will be expanded to a larger scope.

## Figures and Tables

**Table 1:** Summary of commonly used hyperspectral satellites.

Sensor	Spectral Range (nm)	Number of Bands	Spatial Resolution (m)	Width (km)
GF-5	400–2500	330	30	60
ZY-1 02D	400–2500	166	30	60
HysIS	400–2500	326	30	30
PRISSAM	400–2500	239	30	30
EnMAP	420–2450	88	30	30

**Table 2:** Basic information of GF-5 AHSI data.

Hyperspectral Data Identification Number	GF5_AHSI_E78.40_N35.26_20190907_007086_L10000055579
Data quantity	1
Data acquisition time	7 September 2019
Data sources	https://www.cheosgrid.org.cn/index.htm.
Data composition	Visible–near Infrared (VNIR) and shortwave infrared (SWIR)
Band	330
Spatial resolution	30 m

**Table 3:** Basic information of Sentinel-2A AHSI data.

Multispectral Data Identification	S2A_MSIL1C_20211016T052821_N0301_R105_T44SKD
Data quantity	1
Data acquisition time	16 October 2021
Data sources	https://scihub.copernicus.eu/
Data composition	Consisting of spatial resolutions of 10 m, 20 m, and 60 m, respectively
Band	13

**Table 4:** Main parameters of fast line-of-sight atmospheric analysis of spectral hypercubes (FLAASH) atmospheric correction.

Parameter	Value
Scene Center Location	Lat: 35°41′01″ Lon: 78°38′
Sensor Altitude (km)	5.24″
Ground Elevation (km)	705
Pixel Size (m)	5.610
Flight Date	30
Flight Time GMT	7 September 2019
Atmospheric Model	47:7
Aerosol Model	Mid-Latitude Summer
Aerosol Retrieval	Rural

**Table 5:** Relevant setting information.

Parameter	Configuration
GPU	NVIDIA RTX A6000
Epoch	400
Batchsize	1
Optimizer	Adam
Initial learning rate	0.001 initially, linear decay from 50epoch

**Table 6:** Remote sensing image evaluation metric results.

	PSNR	SAM	ERGAS	$Q2^{n}$	$D_{\lambda }$	$D_{s}$	QNR	AG
CNMF	17.534	2.3359	9.7953	0.48969	0.194655	0.4690	0.4276	61.9774
NonRegSRNet	5.7765	3.6498	1.0091	0.02295	0.016447	0.2878	0.7004	20.4492
GSA	21.332	2.1683	5.4638	0.57705	0.194616	0.4690	0.4675	62.0528
HySure	20.176	3.0343	6.8187	0.50037	0.194627	0.4691	0.4275	62.0502
SFIM	20.911	2.2688	5.4639	0.55059	0.194629	0.4691	0.4291	61.9920

**Table 7:** Lithological categories and classification samples of the study area.

Stratigraphic Code	Stratigraphic Name	Lithology	Training Data/ Validation Data (Surface)	Training Data/ Validation Data (Point)
$Qh^{al}$	Quaternary Holocene series	Alluvial sandy soil, gravel–cobble	55	102
$Qh^{3alp}$	Quaternary Upper Pleistocene series	Alluvial–pluvial gravel, sandy soil	91	391
$J_{2}I^{2(mic)}$	Middle Jurassic Longshan Formation Member2	Grayish black, gray microcrystalline limestone	56	121
$J_{2}I1^{\left(cg\right)}$	Middle Jurassic Longshan Formation Member1	purple red, brownish red, gray conglomerate	17	104
$P_{1}s^{3(qzvb)}$	Lower Permian Shenxianwan Formation Member3	Gray white brecciated quartzite	49	282
$P_{1}s^{3(qsd)}$	Lower Permian Shenxianwan Formation Member3	Gray white, gray quartz sandstone	24	134
$P_{1}s^{3(slt)}$	Lower Permian Shenxianwan Formation Member1	Dark gray, grayish green siltstone	110	246
η γ *K*		Monzonitic granite	26	124
VT		Gabbro	90	360
Snow covered area			17	64

**Table 8:** Confusion matrix of user accuracy for each type before and after fusion in lithological classification.

	CNMF	NonRegSRNet	GSA	HySure	SFIM	GF-5	Sentinel-2A
$Qh^{al}$	93.48	79.90	94.24	91.07	91.29	95.45	79.81
$Qh^{3alp}$	91.46	60.49	94.09	94.00	93.05	96.72	60.00
$J_{2}I^{2(mic)}$	93.59	61.32	95.72	92.59	94.00	94.22	61.20
$J_{2}I1^{\left(cg\right)}$	81.95	68.28	93.15	85.57	85.19	78.38	72.92
$P_{1}s^{3(qzvb)}$	69.52	26.23	86.66	79.34	84.89	76.92	24.15
$P_{1}s^{3(qsd)}$	73.04	10.79	85.57	79.27	82.76	80.00	11.18
$P_{1}s^{3(s1t)}$	81.69	51.38	87.04	81.36	84.81	96.27	51.38
η γ K	87.08	70.79	92.80	87.22	85.80	94.05	70.86
VT	80.62	23.68	91.79	81.30	84.60	85.52	22.02
Snow covered area	82.42	83.54	88.11	75.27	87.30	68.41	83.75

## Data Availability

The associated code for this study has been uploaded to Code Ocean. The data presented in this study are available upon request from the corresponding author. The data are not publicly available due to the sensitivity of the information contained therein.
